# OXA-48 Dominance Meets Ceftazidime-Avibactam: A Battle Against Life-Threatening Carbapenem-Resistant Klebsiella pneumoniae Infections in the Intensive Care Unit

**DOI:** 10.7759/cureus.46780

**Published:** 2023-10-10

**Authors:** Uğur Önal, Ülkü Tüzemen, Pınar K Kaya, Remzi İşçimen, Nermin K Girgin, Cüneyt Özakın, Ferda Kahveci, Halis Akalın

**Affiliations:** 1 Infectious Diseases, Uludag University Faculty of Medicine, Bursa, TUR; 2 Microbiology, Uludag University Faculty of Medicine, Bursa, TUR; 3 Anesthesiology and Critical Care, Uludag University, Bursa, TUR; 4 Intensive Care Unit, Uludag University Faculty of Medicine, Bursa, TUR; 5 Anesthesiology and Critical Care, Uludag University Faculty of Medicine, Bursa, TUR; 6 Anesthesiology and Reanimation, Uludag University Faculty of Medicine, Bursa, TUR; 7 Anesthesiology and Reanimation, Uludag University, Bursa, TUR; 8 Medical Microbiology, Uludag University Faculty of Medicine, Bursa, TUR; 9 Infectious Diseases and Clinical Microbiology, Uludag University Faculty of Medicine, Bursa, TUR

**Keywords:** sofa score, ventilatory associated pneumonia, bloodstream infection, carbapenem-resistant klebsiella pneumoniae, ceftazidime-avibactam

## Abstract

Objective

In this study, we aimed to describe the outcomes in ICU patients with bloodstream infection (BSI) or ventilatory-associated pneumonia (VAP) due to carbapenem-resistant *Klebsiella pneumoniae *(CRKP) who received ceftazidime-avibactam treatment at a tertiary care university hospital.

Methods

Patients aged 18 years or older who were admitted to the Anesthesiology and Reanimation ICU at Bursa Uludag University Faculty of Medicine Hospital between June 13, 2021, and July 16, 2023, and diagnosed with BSI or VAP due to CRKP were included in this study.

Results

A total of 42 patients treated with ceftazidime-avibactam were included. Total crude mortality rates were 33.3% on day 14 and 54.8% on day 30. Mortality rates on the 14th and 30th days were 37.5% and 62.5% in patients with BSI and 27.8% and 44.4% in patients with VAP, respectively. There was no statistically significant difference between monotherapy and combination therapy in terms of mortality rates on days 14 and 30, respectively (3/11 vs. 11/31, p=0.620; 5/11 vs. 18/31, p=0.470). Immunosuppression (10/11 vs. 13/31, p=0.005), the Sequential Organ Failure Assessment (SOFA) score ≥8 (at the initiation of treatment; 19/25 vs. 4/17, p<0.001), INCREMENT-CPE score ≥10 (12/16 vs. 3/10, p=0.024) and longer duration (in days) from culture collection to treatment initiation (5.0 ± 0.61 vs. 3.11 ± 0.48, p=0.024) were found to have a statistically significant effect on 30-day mortality. In multivariate analysis, a SOFA score ≥8 at the initiation of treatment (p=0.037, OR: 17.442, 95% CI: 1.187-256.280) was found to be a significant risk factor affecting mortality (30-day).

Conclusion

The mortality rates of patients with CRKP infection who were followed up in the ICU were found to be high, and it was observed that whether ceftazidime-avibactam treatment was given as a combination or monotherapy did not affect mortality. Further multicentre studies with a larger number of patients are needed to gain a comprehensive understanding of the topic, given that this treatment is typically reserved for documented infections.

## Introduction

Infections caused by carbapenem-resistant *Klebsiella pneumoniae* (CRKP) are an important global health problem due to the high mortality rates associated with it [1,2]. CRKP rates have been reported to be 50% or higher in eight countries, including Türkiye, among a total of 45 countries in Europe in 2021 [3]. Moreover, there was a significant upward trend in the prevalence of CRKP infections in Türkiye every year from 2016 to 2021. According to the report of the National Healthcare-Associated Infections Surveillance Network (USHIESA), the overall carbapenem resistance rate in *Klebsiella pneumoniae *isolates in our country was found to be 66.56% in 2022 [4].

While international guidelines recommend the use of new antibiotics such as ceftazidime-avibactam, meropenem-vaborbactam, imipenem-cilastatin-sulbactam, and cefiderocol in the treatment of CRKP infections, the inaccessibility of treatment options such as meropenem-vaborbactam, imipenem-cilastatin-sulbactam, and cefiderocol constitutes a serious problem in our country [5]. In light of this, the aim of this study was to evaluate the efficacy of ceftazidime-avibactam-based treatment regimens and to examine risk factors that may affect the mortality rate of ICU patients with CRKP-related bloodstream infection (BSI) or pneumonia.

## Materials and methods

This study included adult patients (≥18 years of age) who were admitted to the Anesthesiology and Reanimation ICU of Bursa Uludag University Faculty of Medicine Hospital between June 13, 2021, and July 16, 2023, with the diagnosis of BSI or ventilator-associated pneumonia (VAP) due to CRKP. The classification of BSI comprised primary cases (confirmed by blood culture positivity and not originating as a result of infection at another site), or secondary cases, which were inferred to have originated from a site-specific infection in a different area of the body. Pneumonia was defined based on the established criteria, including new or progressing chest infiltrates on radiography, along with at least two of the following indicators: (1) body temperature exceeding 38 °C; (2) declining oxygenation; (3) leukocyte count exceeding 10,000/mm³ or dropping below 4,000/mm³; and (4) the presence of purulent bronchial secretions (leukocyte count >25) and up to 10 epithelial cells in Gram-stained deep endotracheal aspirate (ETA) under 10x magnification. VAP was characterized as pneumonia occurring more than 48 hours post-intubation and initiation of mechanical ventilation. Clinical, demographic, and laboratory findings and antimicrobial treatments in the electronic patient files of the patients were analyzed retrospectively by filling out case evaluation forms.

The inclusion criteria were as follows: (1) ≥18 years of age; (2) a diagnosis of BSI or VAP; (3) a positive culture result for CRKP.

The Acute Physiology and Chronic Health Evaluation II (APACHE II) - a multifactorial risk prediction model based on 12 physiologic measurements, age, and health status for critically ill patients in the ICU - was calculated at the initiation of treatment. In addition, the Sequential Organ Failure Assessment (SOFA) score [0-4 points for each of the Glasgow Coma Scale (GCS) score; PaO_2_/FiO_2_, platelets, bilirubin, urine output, and creatinine levels; use of vasopressor agents; and mean arterial pressure], systemic inflammatory response syndrome (SIRS) [(1) fever >38.0 °C or hypothermia <36.0 °C, (2) tachypnea >20 breaths/minute or pCO_2_ <32 mmHg, (3) tachycardia >90 beats/minute, and (4) leukocytosis >12,000/mm^3^ or leukopenia <4,000/mm^3^], and INCREMENT-CPE (5 points for severe sepsis or septic shock, 4 points for Pitt score ≥6, 3 points for Charlson Comorbidity Index ≥2, and 3 points for bacteremia source other than urinary or biliary tract) scores were calculated at the initiation of treatment [6,7]. Immunosuppression was defined as a history of transplantation, HIV infection, malignancy under active treatment, or drug use (chemotherapy, corticosteroids ≥20 mg prednisone or equivalent daily for at least two or more weeks, calcineurin inhibitors, cytotoxic agents) [8].

*Klebsiella pneumoniae* identification was performed using matrix-assisted laser desorption/ionization time-of-flight mass spectrometry (MALDI-TOF MS). Due to the limited resources at our center, molecular tests like OXA-48 were not carried out. Antimicrobial susceptibility tests were conducted utilizing the outcomes from an automated system (Phoenix™ 100, Becton Dickinson, Sparks, MD). Furthermore, the broth microdilution (Sigma Aldrich, St. Louis, MO) method was applied for assessing colistin resistance. Given the treatment policy restrictions in our country, our study solely included ceftazidime-avibactam-susceptible isolates. Fosfomycin resistance in a total of 11 patients who were included in our retrospective study and whose samples were preserved were also analyzed using the agar dilution method. Antimicrobial susceptibility results were evaluated in accordance with the recommendations of the European Committee on Antimicrobial Susceptibility Testing (EUCAST) [9].

Statistical analyses were performed using IBM SPSS Statistics software version 28.0 (IBM Corp., Armonk, NY). The Student's t-test was used for the analysis of parametric variables and the Mann-Whitney U test was used for nonparametric variables. A p-value below 0.05 in univariate analysis was considered statistically significant and included in binary logistic regression analysis. Binary logistic regression analysis was performed using the "Enter" method.

Ethical committee approval was granted by the Bursa Uludag University Faculty of Medicine Clinical Research Ethics Committee with approval number 2023_17/9.

## Results

A total of 42 patients, 12 of them females, were included in the study. The mean age of the patients was 59.38 ± 3.08 years. While BSI was detected in 24/42 (57%) of the patients, 18/43 (43%) patients were diagnosed with VAP. The most common comorbidities were hypertension (43%), diabetes mellitus (33%), and malignancy (26%). The mean duration of total ICU stay was 57.05 ± 7.58 days. The results of culture antibiograms revealed colistin resistance (39/42, 92.9%), amikacin resistance (15/42, 36%), gentamicin resistance (20/42, 48%), and fosfomycin resistance (16/35, 46% by automated system and 8/11, 73% by agar dilution method).

Mortality rates were 33.3% on day 14 and 54.8% on day 30. Mortality rates on the 14th and 30th days were 37.5% and 62.5% in patients with BSI and 27.8% and 44.4% in patients with VAP, respectively. Monotherapy was preferred in a total of 11 patients (26%). Antibiotics were combined with ceftazidime-avibactam, including polymyxin B or colistin in 12 patients, fosfomycin in 12 patients, amikacin or gentamicin in 14 patients, meropenem in three patients, tigecycline in three patients, and trimethoprim and sulfamethoxazole in one patient. Combination therapy with polymyxins was particularly preferred for the cases with polymicrobial culture positivities (especially for *Acinetobacter* spp.) based on sensitivity results. Moreover, aminoglycosides and/or fosfomycin combinations were the preferred option for patients experiencing septic shock, those undergoing continuous renal replacement therapy (CRRT), and individuals diagnosed with VAP. The mortality rates for patients receiving combination therapy were 35.5% on the 14th day and 58.1% on the 30th day. For patients receiving monotherapy, the rates were 27.3% on the 14th day and 45.5% on the 30th day. There was no statistically significant difference between monotherapy and combination therapy in terms of 14th- and 30th-day mortality rates, respectively (3/11 vs. 11/31, p=0.620; 5/11 vs. 18/31, p=0.470). Whether the combination therapy included fosfomycin (8/12 vs. 15/30, p=0.327), colistin/polymyxin B (8/12 vs. 9/17, p=0.460), or aminoglycoside (6/13 vs. 11/16, p=0.219) had no significant effect on 30-day mortality rates. The mean duration of treatment with ceftazidime-avibactam was 9.76 ± 0.81 days.

Immunosuppression (10/11 vs. 13/31, p=0.005), SOFA score ≥8 at the start of treatment (19/25 vs. 4/17, p<0.001), INCREMENT-CPE score ≥10 (12/16 vs. 3/10, p=0. 024) and a longer duration (in days) from culture collection to the start of treatment (5.0 ± 0.61 vs. 3.11 ± 0.48, p=0.024) were found to have a statistically significant effect on 30-day mortality (Table 1).

**Table 1:** Assessment of risk factors for 30-day mortality based on chi-square and t-test analysis *Acinetobacter baumannii (n=10), *Pseudomonas aeruginosa* (n=5), *Staphylococcus epidermidis *(n=2), *Klebsiella aerogenes *(n=1), *Citrobacter koseri* (n=2), *Stenotrophomonas maltophilia *(n=2), *Escherichia coli *(n=1), *Staphylococcus aureus *(n=1), *Staphylococcus haemolyticus *(n=1) SD: standard deviation

Variables		30-day mortality		P-value
		Yes	No	
Gender, n (%)	Female	7 (58%)	5 (42%)	0.769
	Male	16 (53%)	14 (47%)	
Age, years, mean ± SD		61.09 ± 3.82	57.32 ± 5.08	0.549
Hypertension, n (%)	Present	8 (44%)	10 (56%)	0.245
	Absent	15 (63%)	9 (37%)	
Diabetes mellitus, n (%)	Present	9 (64%)	5 (36%)	0.381
	Absent	14 (50%)	14 (50%)	
Chronic renal failure, n (%)	Present	6 (86%)	1 (14%)	0.071
	Absent	17 (49%)	18 (51%)	
Chronic obstructive pulmonary disease, n (%)	Present	4 (57%)	3 (43%)	0.890
	Absent	19 (54%)	16 (46%)	
Immunosuppression, n (%)	Present	10 (91%)	1 (9%)	0.005
	Absent	13 (42%)	18 (58%)	
The Systemic Inflammatory Response Syndrome (SIRS) score ≥2, n (%)	Present	13 (59%)	9 (41%)	0.554
	Absent	10 (50%)	10 (50%)	
The Sequential Organ Failure Assessment (SOFA) score ≥8, n (%)	Present	19 (76%)	6 (24%)	<0.001
	Absent	4 (24%)	13 (76%)	
The Acute Physiology And Chronic Health Evaluation (APACHE) II score ≥15, n (%)	Present	22 (58%)	16 (42%)	0.209
	Absent	1 (25%)	3 (75%)	
INCREMENT-CPE score ≥10, n (%)	Present	12 (75%)	4 (25%)	0.024
	Absent	3 (30%)	7 (70%)	
Bloodstream infection, n (%)	Present	15 (63%)	9 (37%)	0.245
	Absent	8 (44%)	10 (56%)	
Combination treatment, n (%)	Present	18 (58%)	13 (42%)	0.470
	Absent	5 (46%)	6 (54%)	
Polymicrobial infection*, n (%)	Present	9 (41%)	13 (59%)	0.059
	Absent	14 (70%)	6 (30%)	
Duration from collection of index culture to initiation of treatment, days, mean ± SD		5.0 ± 0.61	3.11 ± 0.48	0.024

Variables with a p-value below 0.05 in univariate analysis [immunosuppression, SOFA score ≥8 (at the initiation of treatment) INCREMENT-CPE score ≥10, and duration from collection of index culture to initiation of treatment (in days)] were included in binary logistic regression analysis. A SOFA score ≥8 at the initiation of treatment (p=0.037, OR: 17.442, 95% CI: 1.187-256.280) had a significant effect on 30-day mortality in multivariate logistic regression analysis (Table 2).

**Table 2:** Risk factors for 30-day mortality in multivariate logistic regression analysis Nagelkerke R^2^: 0.614

Variables	P-value	Odds ratio	95% CI
Immunosuppression	0.464	-	-
The Sequential Organ Failure Assessment (SOFA) score ≥8 (at the initiation of treatment)	0.037	17.442	1.187-256.280
INCREMENT-CPE score ≥10	0.105	-	-
Duration from collection of index culture to initiation of treatment in days	0.118	-	-

## Discussion

Due to limited treatment options and high mortality rates, the management of CRKP infections is of vital importance, especially in intensive care patients, and we believe that our study contributes to the literature on the use of ceftazidime-avibactam in the treatment of CRKP infections in our country.

In a multicenter, prospective, observational study from Greece, a total of 147 patients (KPC+ in 140 patients, OXA-48+ in seven patients) with CRKP infection (64.6% with BSI) were examined. The 28-day mortality was found to be significantly lower in the patient group (n=71) who received ceftazidime-avibactam treatment for CRKP-related BSI compared to those who did not receive ceftazidime-avibactam (n=71) (18.3% vs. 40.8%, p=0.005) [10]. Van Duin et al. analyzed a total of 137 patients with carbapenem-resistant Enterobacteriaceae (CRE) infection (46% of whom had BSI); 133 of them had CRKP, 38 patients received ceftazidime-avibactam, and 99 patients received colistin treatment. The 30-day mortality was found to be significantly lower in patients receiving ceftazidime-avibactam treatment compared to patients receiving colistin (8% vs. 33%, p=0.001) [11]. In a study where 42 of a total of 90 patients with CRKP infection received ceftazidime-avibactam treatment, the 30-day mortality rate was found to be 19%, and ceftazidime-avibactam treatment was found to be superior to other treatments such as colistin, tigecycline, and fosfomycin in terms of efficacy, especially in critically ill patients (6/29 vs. 13/29, p=0.05) [12]. In addition, in a study evaluating a total of 77 ICU patients with CRE infection, 41 patients received ceftazidime-avibactam treatment, and the ICU survival rate was found to be higher in this group compared to colistin-containing combination treatments (61% vs. 41.7%). In 22 patients with BSI who received ceftazidime-avibactam treatment, the ICU survival rate was 59.1% [13].

Ceftazidime-avibactam is among the commonly recommended treatments for CRE infections and it is cost-effective in the treatment of BSI and pneumonia due to CRE [14]. A comparative study of ceftazidime-avibactam and meropenem-vaborbactam treatments for CRE infections revealed that among the 15 patients receiving ceftazidime-avibactam, resistance emerged in three individuals (20%) whose infections recurred within 90 days [15]. In our country, the use of ceftazidime-avibactam has been restricted in the treatment of documented infections in accordance with the rules of the Social Security Institution Communiqué on Healthcare Practices to be initiated in second and/or third-level intensive care patients who have been proven to be sensitive to ceftazidime-avibactam in vitro [16].

Feng et al. evaluated a total of 178 Enterobacterales isolates by using a rapid ResaCeftazidime-avibactam NP test where susceptibility results for ceftazidime-avibactam could be obtained within 4.5 hours and found 99% category agreement and 0% very major errors for the rapid test they applied in *Klebsiella pneumoniae* samples [17]. A total of 137 patients with CRE (65% KPC+) BSI were analyzed in a study by Ackley et al., and it was reported that the time to initiation of ceftazidime-avibactam treatment (median: 24 vs. 50 hours, p=0.009) and 30-day mortality (24% vs. 47%, p=0.007) were lower in patients (n=51) who underwent blaKPC polymerase chain reaction (PCR) testing in blood cultures compared to other patients (n=86) [18]. Falcone et al. conducted a study involving 102 patients with CRKP BSI and reported a 30-day mortality rate of 45%. The research also demonstrated that patients who survived had a shorter median time to appropriate antibiotic therapy [8.5 hours (IQR: 1-36)] compared to those who did not survive [48 hours (IQR 5-108), p=0.014]. They also showed that receiving in vitro active therapy within 24 hours of blood culture collection was associated with a decreased 30-day mortality (HR: 0.36, 95% CI: 0.188-0.690, p=0.0021) [19].

In our study, the overall 30-day mortality was 54.8%, while in patients with BSI, it was 62.5%. Mortality was found to be higher in patients with longer duration (days) from culture collection to the start of treatment (5.0 ± 0.61 vs. 3.11 ± 0.48, p=0.024) in our study. The elevated mortality rates in comparison to those in the literature could be attributed to our study's exclusive focus on ICU patients and the limited application of ceftazidime-avibactam treatment solely to documented infections, in accordance with the restricted usage protocol in our country. Therefore, we believe that there is a need for devising rapid methods that can provide earlier results in the diagnostic process and resistance tests. Table 3 presents various studies in the literature exclusively focusing on ceftazidime-avibactam treatment for CRKP infections in ICU patients.

**Table 3:** Literature review of ceftazidime-avibactam treatment for CRKP infections in ICU patients Values presented as mean ± standard deviation or median (IQR) *28-day mortality rate ICU: intensive care unit; SOFA: Sequential Organ Failure Assessment; APACHE II: Acute Physiology and Chronic Health Evaluation II; CRKP: carbapenem-resistant *Klebsiella pneumoniae*;* *VAP: ventilatory-associated pneumonia; HAP: hospital-acquired pneumonia; BSI: bloodstream infection; N/A: not available

Study	Total number of ICU patients	Type of infection	SOFA score at the onset of infection	APACHE II score at the onset of infection	30-day mortality rate
Zheng et al. [20]	62	All CRKP infections	N/A	17.5 (14.8-20)	33.9%
Shi et al. [21]	43	VAP or HAP	7 (3-10)	12 (10-16)	30.2%*
Tsolaki et al. [13]	41	All CRKP infections	7.90 ± 0.47	19.11 ± 1.08	14.6%
Falcone et al. [19]	13	BSI	N/A	N/A	23.1%
Our study	42	BSI or VAP	9.05 ± 0.6	24.74 ± 1.0	54.8%

In a retrospective multicenter study, a total of 244 ICU patients with CRKP infection were examined and it was shown that an APACHE II score ≥15 was a significant risk factor for mortality (aOR: 1.88, 95% CI: 1.29-4.06, p=0.034) [22]. In our study, although the 30-day mortality rate was higher in patients with an APACHE II score ≥15, the difference was not statistically significant (p=0.209). In a retrospective study from Greece evaluating the INCREMENT-CPE score in ICU patients with CRKP bacteremia, a total of 384 patients were analyzed, and 14-day mortality was found to be 26.3%. An INCREMENT-CPE score of ≥10 demonstrated a sensitivity of 98% and a negative predictive value of 98.7%. This scoring system exhibited comparable predictive efficacy for mortality with Simplified Acute Physiology Score II (SAPS II), SOFA, and Pitt bacteremia scores. Notably, in the same study, the SOFA score ≥8 points presented the highest area under the curve value (0.815) among the scoring systems [7].

In our study, a prognosis threshold of 10 points or higher was applied to the INCREMENT-CPE score, and 30-day mortality was found to be higher in patients with INCREMENT-CPE score ≥10 (12/16 vs. 3/10, p=0.024). Nevertheless, this statistically significant difference did not maintain significance in the multivariate analysis. In addition, a SOFA score ≥8 at the initiation of treatment had a significant effect on 30-day mortality in multivariate logistic regression analysis (p=0.037, OR: 17.442, 95% CI: 1.187-256.280). In terms of 30-day mortality among ICU patients, a high SOFA score was a significant risk factor, in line with the data in the literature. Furthermore, we believe that the INCREMENT-CPE score was solely measured in patients with BSI (n=26) constitutes a significant factor contributing to the lack of significance in the multivariate analysis.

In a retrospective study involving 62 critically ill patients with CRKP infection in China, 41 individuals (66.1%) were administered ceftazidime-avibactam combination therapy (with an antimicrobial therapy that was found to be resistant in vitro), while 21 patients (33.9%) received ceftazidime-avibactam monotherapy. The 30-day mortality rates were 24.4% (10/41) for the combination therapy group and 47.6% (11/21) for the monotherapy group (p=0.028) [22]. In a multicenter retrospective observational study from Italy by Tumbarello et al., a total of 577 patients (391 with BSI) with CRKP infection who were treated with ceftazidime-avibactam were examined, and no significant difference was found in terms of 30-day mortality between patients treated with ceftazidime-avibactam as monotherapy (n=165) and those treated with combination therapy (n=412) (26.1% vs. 25.0%, p=0.79) [23]. In a systematic review and meta-analysis evaluating patients with CRE infection receiving ceftazidime-avibactam treatment (n=503), no difference in mortality rate was observed in patients receiving ceftazidime-avibactam combination therapy compared to monotherapy (OR: 0.96, 95% CI: 0.65-1.41) [24]. In our study, no statistically significant difference was found between monotherapy and combination therapy in terms of mortality rates on days 14 and 30, respectively (3/11 vs. 11/31, p=0.620; 5/11 vs. 18/31, p=0.470).

This study has a few limitations, primarily related to its single-center, retrospective design and the fact that it included a relatively small number of. Due to the small sample size, we were unable to conduct a Cox regression analysis or compare the clinical characteristics of patients treated with combination therapy versus monotherapy in our study. Moreover, it is important to acknowledge other limitations as well, such as potential bias related to mortality due to variations in time to treatment. Finally, carbapenemase typing was not performed in our study; however, in a recent study conducted at our center, the enzyme distribution in CRKP isolates was shown to be as follows: OXA-48: 49.1%, KPC: 29.1%, OXA-48 + KPC: 10.9%, NMD: 3.6%, NDM + OXA-48: 1.8%, NDM + OXA-48 + KPC: 1.8%, and NDM + VIM: 1.8% [25].

## Conclusions

In our study, mortality rates of patients with CRKP infection who were followed up in the ICU were found to be high, and whether ceftazidime-avibactam treatment was given as a combination or monotherapy did not affect mortality. Mortality was found to be higher in patients with a longer duration (in days) from culture collection to treatment initiation. Multivariate logistic regression analysis revealed that a SOFA score ≥8 at the initiation of treatment was a significant risk factor in terms of 30-day mortality.
